# Predicting Hair Cortisol and Cortisone Concentration in Postpartum Women through Repeated Measurements of Perceived Stress

**DOI:** 10.3390/metabo11120815

**Published:** 2021-11-29

**Authors:** Jessica Lang, Susanne Stickel, Petra M. Gaum, Ute Habel, Jens Bertram, Simon B. Eickhoff, Natalia Chechko

**Affiliations:** 1 Institute for Occupational, Social and Environmental Medicine, Medical Faculty, RWTH Aachen University, Pauwelsstraße 30, 52074 Aachen, Germany; pgaum@ukaachen.de (P.M.G.); jbertram@ukaachen.de (J.B.); 2 Department of Psychiatry, Psychotherapy and Psychosomatics, Medical Faculty, RWTH Aachen University, Pauwelsstraße 30, 52074 Aachen, Germany; sstickel@ukaachen.de (S.S.); uhabel@ukaachen.de (U.H.); nchechko@ukaachen.de (N.C.); 3 Institute of Neuroscience and Medicine: JARA-Institute Brain-Structure-Function Relationship (INM 10), Research Center Jülich, Wilhelm-Johnen-Straße, 52425 Jülich, Germany; 4 Institute of System Neuroscience, Medical Faculty, Heinrich Heine University Düsseldorf, Moorenstraße 05, 40225 Düsseldorf, Germany; s.eickhoff@fz-juelich.de; 5 Institute of Neuroscience and Medicine: Brain and Behavior (INM 7), Research Center Jülich, Wilhelm-Johnen-Straße, 52425 Jülich, Germany

**Keywords:** cortisol, hair cortisone, postpartum women, stress experience

## Abstract

To investigate whether hair cortisol (HCC) and hair cortisone (HCNC) can be predicted by repeated stress reports from postpartum women in different mental health conditions (non-depressed, ND, adjustment disorder, AD, postpartum depression, PPD), 240 mothers (mean age 31.8 years; SD = 4.7) were monitored from within 1 to 6 days of childbirth over a period of three months. HCC and HCNC in 3 cm hair samples were assessed via triple mass spectrometry after liquid chromatographic separation. Every second day, participants reported their stress levels online. The summed perceived stress scores were not found to be predictive of HCC. However, perceived stress predicted a decrease in HCNC (r_Spearman_ = –0.153, *p* = 0.035) and an increase in the HCC/HCNC ratio (r_Spearman_ = 0.304, *p* < 0.001) in the ND group. With AD in the first few weeks after childbirth, an inverse effect appeared for HCNC (r_Spearman_ = 0.318, *p* = 0.011), suggesting an overall downregulation of the HPA axis owing to the stressful experience of adjusting to the new situation. No effects were found for mothers developing PPD. The indirect results of HPA-axis activity are better indicators of the experience of psychological stress in postpartum women than the absolute HCC value.

## 1. Introduction

Pregnancy and childbirth are life events that require extensive adjustments [1]. Stress exposure during pregnancy and after childbirth has been linked to mental and somatic illnesses for mothers and their infants [2,3]. Thus, it is important to investigate the impact of mothers’ perceived stress and the physiological stress response. In response to stress, the activated hypothalamus–pituitary–adrenal (HPA) axis releases cortisol. However, in a recent meta-analysis, the overall meta-analytic correlations failed to show a link between perceived stress measures and hair cortisol concentrations (HCC) [4]. Only Kalra and colleagues found a correlation between HCC and stress reports in pregnant women [5], while the majority of other studies (some involving larger samples of healthy pregnant women or women after childbirth) were unable to replicate the findings [6,7]. Additionally, the consideration of both cortisol and cortisone as stress biomarkers in pregnant women were suggested to be more meaningful. For instance, Scharlau and colleagues found an association between cortisol/cortisone metabolism (not cortisol value alone) and self-reported chronic stress in the 2nd and 3rd trimesters of pregnancy [3].

In addition, the overall lack of association between the psychological and physiological stress responses was attributed to the retrospective assessment methods with the potential of recall bias for stress reports [4], leading researchers to suggest a repeated measurement approach [6,8].

The goal of the present study was to link repeated online assessments of chronic stress experience to parallel assessments of hair cortisol and cortisone in postpartum women. We hypothesized that (H1) the more mothers report perceived stress during the three-month postpartum period, the higher their HCC levels would be, indicating an increased cortisol secretion under conditions of higher perceived stress, which (H2) would lead to lower HCNC values, indicating a reduced conversion of cortisol in its inactive form. Finally, the more perceived stress mothers report, the higher their HCC/HCNC ratio would be, indicating a generally increased conversion of HCNC into HCC under high-stress (H3) conditions.

Given that the meta-analytic findings indicated different associations of HCC with different strain-exposed subgroups [4], our comparisons involved, in addition to healthy women (ND), women who developed postpartum depression (PPD), which affects up to 11% of mothers [9], and those with postpartum adjustment disorder (AD), which is characterized by disproportionate levels of distress following an identifiable psychological stressor (i.e., childbirth), although the symptoms of this condition do not meet the criteria for major depression [10].

## 2. Results

### 2.1. Sample Description

Please see Table 1 for the descriptive information of the study sample concerning the variables of interest. Other sample characteristics (demographic data and obstetric, child-related and anamnestic information) are provided in the Supplementary Materials (Table S1). The average start of participation was 2.75 days (SD = 1.55) after childbirth.

Over the course of 12 weeks, the three groups differed significantly in the subjectively perceived stress experience (all *p* < 0.001), with PPD having the highest stress values compared with AD (d = 0.96) and ND (d = 1.42), and AD having higher values than ND (d = 0.55). In order to carry out additional group comparisons, the subjective stress values were cumulated at an interval of three weeks. While ND on average had significantly lower stress levels than PPD at all times (all *p* < 0.001; all d = 0.89–1.45), the AD group showed elevated levels of stress only in the first 6 weeks compared with ND (all *p* < 0.01, all d = 0.66–0.68). In weeks 6–12, the AD group showed no difference to ND, but significantly lower values than PPD (all *p* < 0.001, all d = 0.86–1.36).

Compared with ND, the AD and PPD groups had significantly higher EPDS scores immediately after childbirth (ND vs. AD: *p* < 0.001, d = 1.01, ND vs. PPD: *p* < 0.001, d = 0.57) and 3 weeks postpartum (ND vs. AD: *p* < 0.001, d = 1.94, ND vs. PPD: *p* < 0.001, d = 1.69). After 6, 9 and 12 weeks, the EPDS scores in the AD group were significantly lower compared with the PPD group (all *p* < 0.001, d_6weeks_ = 0.77, d_9weeks_ = 1.25, d_12weeks_ = 1.86) but were still higher compared with the ND group (all *p* < 0.001, d_6weeks_ = 1.59, d_9weeks_ = 0.126, d_12weeks_ = 1.49). Women in the ND group experienced fewer stressful life events compared with AD (*p* = 0.008, d = 0.32) and ND (*p* = 0.008, d = 0.37). 

With regard to glucocorticoid concentration at T0 and T1 (see Table 1), no significant differences were detected between the groups for HCC. However, at T1, there was a significant difference in HCNC between the groups and the HCC/HCNC ratio at T1 was significantly higher in the ND than in the AD group (*p* = 0.014, d = 0.19).

### 2.2. Stress and the Association with Glucocorticoid Concentration

The first column of Table 2 contains the results of Hypotheses 1–3 for mentally healthy mothers. Hypothesis 1 could not be confirmed in that there was no significant association between the sum of perceived stress over a period of three months and HCC. Hypothesis 2 was confirmed based on a significant negative correlation between reports of elevated perceived stress and HCNC at T1 (*p* = 0.035), indicating a reduced conversion of HCC into HCNC under high-stress conditions. Similarly, stress reports were positively associated with an increase in the HCC/HCNC ratio, indicating an increased conversion of HCNC to HCC under high-stress conditions and thus confirming hypothesis 3. 

The research question for the AD and PPD groups provided the following results (see Table 2 columns 2 and 3). In the AD group, a significant positive correlation occurred for HCNC at T1 (*p* = 0.011), indicating an increased conversion of HCC into HCNC. For women who had developed a PPD, the associations between the hair steroid levels and perceived stress were not significant.

**Figure 1:**
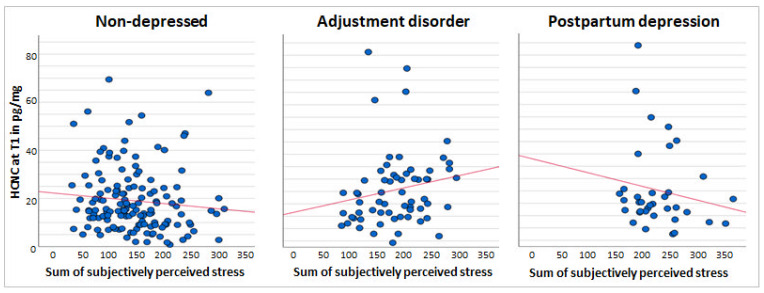
Scatter plots across all the groups of the sum of subjectively perceived stress and raw values of HCNC (in pg/mg) in the three months postpartum (T1), with an adjustment line.

**Figure 2:**
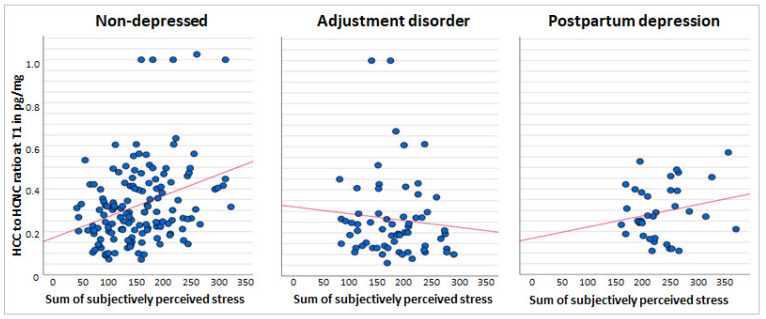
Scatter plots across all the groups of the sum of subjectively perceived stress and raw values of the HCC/HCNC (hair cortisol to hair cortisone concentration) ratio (in pg/mg) in the three months postpartum (T1), with an adjustment line.

Figure 1 and Figure 2 visualize the association between the sum of perceived stress over the course of three months postpartum and the glucocorticoid levels at T1 for HCNC and HCC/HCNC ratio, respectively. The scatter plots depict the differences in the range of perceived stress between the three groups of mental health states in postpartum mothers, showing the largest range in the ND group and smaller ranges in the AD and PPD groups. The PPD stress reports are on the right side of the distribution, indicating an overall higher stress perception. 

## 3. Discussion

In our study, we found a distinct association between perceived stress and hair glucocorticoid levels in the three participant subgroups. The more the non-depressed women perceived stress after childbirth, the lower their HCNC values were found to be, and the greater their HCC/HCNC ratio during the first three months postpartum. These findings corresponded to an expected increase in the conversion of cortisone to its active form of cortisol under increased stress. We observed this effect despite the natural decline in HCC in the days and weeks following delivery [3,11]. In line with most previous studies, no association was found between HCC levels and subjectively reported stress experiences [4].

One reason for the lack of association in absolute physiological outcomes may be the individual differences in the physiological stress reactivity [12]. There is a large variation across individuals with respect to their physiological response to otherwise equivalently reported perceived stress [13]. Likewise, the range of hair steroid levels reported in the current and previous studies is very large, lacking in overall reference values of clinical relevance. Therefore, a higher individual cortisol value may not necessarily determine more subjectively experienced stress compared with another individual.

Corroborating the conclusion drawn by Scharlau et al. [3], our results indicate that in women three months after childbirth, HCC alone is not a sufficient marker for psychological stress assessment. Given the additional physiological peculiarities of pregnancy and childbirth, the inactive form of HCNC and the HCC/HCNC ratio should also be measured. 

Interestingly, for HCNC, the effects were opposite in women who experienced AD after childbirth. The stress levels and EPDS scores were the highest in the first six weeks postpartum, with scores in the first three weeks comparable with PPD and scores in the last six weeks of the study comparable with ND. According to previous research, these types of physiological effects represent an overall downregulation of the HPA axis after past stressful experiences [14]. In the AD group, the endocrine response appeared to have been reduced after the women were able to successfully adjust themselves to the new situation. A more precise assessment in the AD group could have been possible with 4- to 6-week intervals of cumulative cortisol release. 

The association within the PPD group did not reach statistical significance, due likely to the relatively small sample size of that group. Only 9% of our overall sample developed a PPD within the study period [15]. This prevalence corresponds to those reported in the literature [9]. In addition, the participants diagnosed with PPD continuously reported elevated levels of stress over the entire observational period, which was in line with studies related to MDD [16] but restricted the variable’s range. Additionally, they did not show the typical decrease in glucocorticoids (see Supplementary Materials) in the postpartum period, which might have hindered these mothers from hormonally adjusting to the new situation. 

Theories related to stress consequences assume a strong association between physiological stress responses (e.g., the expression of stress hormones) and psychological stress experience (e.g., self-reported perception of stress). It is this association that is referred to as psychoendocrine covariance [8]. However, psychological reactions to acute stress are more dynamic than the reaction of the hormone system, explaining the so-called lack of psychoendocrine covariance between psychological and physiological responses [8]. To circumvent the different dynamic processes, the present study matched stress assessment and the accumulated hair glucocorticoid levels over the same time period.

In doing so, we also circumvented the methodological challenge to reduce retrospective biases in recall of past stress experiences at just one or a few time points [17,18]. The retrospective nature of psychological stress assessment may also lead to social desirability biases, as discussed in a review by Stalder and Kirschbaum [19]. In addition to repeated assessments, an alternative solution may be to operationalize the psychological assessment of stress more objectively. For example, Weckesser et al. [20] reported an effect regarding the frequency of potential weekly hazards, even though they did not find an association between the perceived stress measured monthly and hair glucocorticoid concentration in their study. This more objective operationalization of stress, by asking individuals about the countable occurrence of specific stressors, may allow for a more unbiased assessment of the individual psychological stress experience.

One strength of our study was the sample size and the assessment of HCC and HCNC in a group with similar age and life stages. With past studies having been criticized for their use of low-stress samples [4], the inclusion of non-depressed women, who could also be vulnerable to heightened levels of stress on account of childbirth [21], was an additional strength of our study. In fact, our data show a wide range of stress levels, especially in the non-depressed sample. Nevertheless, with respect to the generalizability and comparability of the findings, it needs to be borne in mind that significant differences in HCC between men and women have been reported, with men showing higher HCC than women [4,22].

In addition to these potential gender differences, there are currently no clinical standards or normative values for HCC [23], due to the different hair analysis methods (immunoassay techniques vs. liquid chromatography coupled to tandem mass spectrometry LC/MS) employed for steroid assessment [24]. Given that immunoassay techniques have been seen to consistently overestimate the raw HCC concentrations due to potential cross-reactions of antibodies with other steroids in the hair matrix, it is difficult to compare the HCC values across studies [25]. Unlike our study, the three studies mentioned above found an association between self-reported stress and HCC using immunoassay techniques instead of LC/MS. Additionally, the lack of standardization in the measurement methods used in those studies renders generalization of the results and general comparability of HCC more difficult [24].

Another problem with hair analysis is the potential impact on steroid assessment when participants report treatments such as hair dying. Single studies have shown reductions in hair cortisol concentration or the HCC/HCNC ratio as a potential consequence of hair dying, although the results are inconsistent. Nonetheless, HCC is generally regarded to be a relatively robust marker with respect to specific hair characteristics following chemical treatments [26]. Since only 18 of our participants in the final sample had dyed hair, we were unable to statistically control for hair treatment. However, these women did not have particularly low glucocorticoid levels. Thus, the potential for a bias in the results of the present study appears to have been minimal.

## 4. Materials and Methods

### 4.1. Procedure

Data collection for the present study occurred within the ongoing longitudinal RiPoD study (Risk for Postpartum Depression (RiPoD) aimed at early recognition of PPD. Beginning within one to six days of childbirth (time point T0), a large cohort of postpartum mothers was recruited at the study center between November 2015 and December 2020. Following informed consent, the participants were initially screened for signs of prenatal depression and were only included in the study if not diagnosed with clinical depression at the time of childbirth. At T0, the first hair sample was taken and the clinical–anamnestic screenings (demographic information, information about the pregnancy as well as individual and family psychiatric history) were carried out. The participants were screened for postpartum depressive symptoms by means of an online survey software (“Survey Monkey”, https://www.surveymonkey.de/, accessed on 28 November 2021) at several time points (3 weeks, 6 weeks and 9 weeks postpartum). In addition, the subjectively perceived stress level was queried online every two days, to facilitate a continuous mapping of the individual stress experience over the entire observation period. After the 12-week period (T1), a second hair sample was taken (on average M = 86.36 days postpartum, SD = 8.40), depressive symptoms were assessed and a clinical interview was conducted by an experienced psychiatrist (NC). Based on the DSM-5 criteria, participants with depressive mood were assigned to either the PPD group or the AD group, and those without depressive symptoms to the non-depressed (ND) group. 

The study protocol was in accordance with the Declaration of Helsinki and approved by the Institutional Review Board of the study center.

### 4.2. Participants

In total, 617 women were recruited at the obstetrics ward of the study center within the time frame mentioned above. The exclusion criteria with respect to the mother’s health status were the following: depression or any other manifest psychiatric condition at the time of recruitment, severe birth- and pregnancy-related complications (e.g., eclampsia, HELLP), alcoholic or psychotropic substance dependency or use during pregnancy, history of psychotic or manic episodes, antidepressant or antipsychotic medication during pregnancy, and lack of sufficient command of German or English. The exclusion criteria based on the child’s health condition comprised very premature birth (less than 29 weeks of gestation), genetic defects (e.g., trisomy) or pathological assessment on the basis of the German Child Health Test (U2). 

Of the 617 screened participants, 358 were excluded from analysis because no (or an insufficient amount of) hair could be obtained at T0 or T1 for glucocorticoid measurement, incomplete follow-up data, or because of significant outlying values calculated with Cook’s distance. Another 4 women were excluded due to postpartum development of panic disorder or anxiety symptoms, and 16 women, who were not depressed at follow-up, were excluded because of slightly elevated self-reported depression scores during the three postpartum months and thus could not be clearly assigned to any of the defined study groups.

The final number of participants available for further analysis was 240. Of these, 141 remained non-depressed during the postpartum period, while 63 developed AD and 36 developed PPD. 

### 4.3. Patient Involvement Statement

At the time of the study, no patients or patient organizations were involved.

### 4.4. Questionnaires

Perceived stress: Every second day (45 days), following commencement of the 90-day study period, the participants received an email link through which they could log into the online survey. Mothers were asked to rate their stress level on a response scale from 1 (indicating low levels of stress) to 10 (high levels of stress). The self-constructed stress-related statement the participants were required to respond to was as follows: “For the last two days, I have felt extremely stressed” (representing their stress experience). In 45 days, on average we collected 39.6 (SD = 3.4) responses per participant. As HCC and HCNC represent the accumulated concentration of cortisol and cortisone in the hair over a period of 3 months, the sum variable of stress as the accumulated subjective stress experience was used as the equivalent variable.

Group Categorization: The Edinburgh Postnatal Depression Scale (EPDS) was used as a self-report instrument for the screening of PPD [27]. In addition, we relied on the Structured Clinical Interview for DSM V [28], which was conducted by an experienced psychiatrist (N.C.) during T0 to guarantee that only psychologically healthy mothers were included in the study.

### 4.5. Sample Collection and Preparation

The average hair growth is considered to be 1 cm/month, with the posterior vertex region of the head showing the least variation in growth rates [29]. Therefore, the hair samples were taken shortly after delivery (1–6 days postpartum, T0) and 12 weeks postpartum (T1) from as close to the scalp as possible at the posterior vertex of the head. The first hair sample at T0 reflected cortisol and cortisone expression over the last trimester of pregnancy, and the second hair sample at T1 reflected the three months postpartum (the period during which self-reported stress levels were also routinely monitored). Hair samples were stored in aluminum foil and were treated as described in Quinete and colleagues [30]. In brief, 3 cm of hair was cut, and approximately 50 mg was weighed in a polypropylene sampling tube using a microbalance. Hair samples were washed briefly with 2-propanol, extracted with a 4-fold deuterium isotope-labeled internal standard of cortisol and methanol for 24 h and then analyzed with liquid chromatography triple quadrupole mass spectrometry using an ion trap (Agilent Technologies 1200 infinity series—QTRAP 5500 ABSciex, Darmstadt, Germany). For both cortisol and cortisone, the limit of quantification in the extracted hair matrix was 0.05 ng/mL (or 2 pg/mg hair).

### 4.6. Statistics

The statistical analyses were performed using SPSS^®^ 25.0 [31] for Windows^®^. Testing the HCC and HCNC data by means of the Shapiro–Wilk test revealed a positively skewed distribution. Therefore, non-parametric tests were conducted.

Before testing the hypotheses, the ratio of HCC and HCNC was calculated by dividing HCC with HCNC. In general, a ratio higher than 1 means a reduced conversion of HCC to HCNC, while an HCC/HCNC ratio lower than 1 indicates an increased conversion of cortisol to cortisone. The higher the HCC/HCNC ratio, the greater the conversion of cortisone into cortisol, representing stress exposure.

In order for an accurate description of the three subgroups of mental health states, we calculated the analysis of variance (ANOVA) pertaining to the sample differences in perceived stress, depressivity and HCC and HCNC levels. In cases of a violation of the assumption of homogeneity of variance (Levene’s test), Welch’s F test was performed. The significant findings were pursued with Games–Howell-corrected pairwise comparisons. 

Spearman’s rank correlations were used to determine whether the sum of perceived stress was associated with HCC, HCNC and HCC/HCNC ratio at T1, applying one-sided tests for the hypotheses and two-sided tests for the research questions.

The effect sizes of the significant results are reported using partial eta squared (ηp^2^) for F-tests (small: 0.020–0.059; medium: 0.06–0.139; large: 0.14 and greater) and Cohen’s d for pairwise comparisons (small: 0.20–0.49; medium: 0.50–0.79; large: 0.80 and greater [32]).

## 5. Conclusions

Our findings indicate that, as an indirect stress parameter, HCNC is a potential biomarker not only for physiological stress reactions in the postpartum period but also for psychologically perceived stress. Subjectively perceived chronic stress accompanies an increased conversion of cortisone to its biologically active form of cortisol, rendering the relative changes in steroid concentrations more relevant than their absolute levels. Future research should further the assessment of the impact of psychological and physiological stress responses specifically in the context of PPD. The follow-up care of postpartum mothers should include stress assessment to help prevent potentially adverse mental and somatic health outcomes that can also affect the health and well-being of the newborn.

## Figures and Tables

**Table 1:** Sample characteristics of the study population.

	Non-Depressed	Adjustment Disorder	Postpartum Depression	Statistics
	Mean (SD)	Mean (SD)	Mean (SD)	
Age	32.01 (4.45)	32.25 (4.91)	30.69 (5.22)	F (2, 239) = 1.41, *p* = 0.247
Perceived stress sum	152.19 (62.39)	184.65 (55.19)	234.64 (50.87)	F (2, 239) = 29.66, *p* < 0.001 ^a, b, c^
0–3 weeks	42.98 (17.69)	55.63 (19.56)	57.53 (15.89)	F (2, 239) = 16.17, *p* < 0.001 ^a, b^
3–6 weeks	40.02 (17.23)	51.06 (16.47)	61.33 (17.19)	F (2, 239) = 26.04, *p* < 0.001 ^a, b, c^
6–9 weeks	37.65 (19.27)	43.47 (17.03)	58.19 (17.99)	F (2, 239) = 17.82, *p* < 0.001 ^b, c^
9–12 weeks	31.52 (17.87)	34.48 (15.93)	57.58 (19.57)	F (2, 240) = 32.52, *p* < 0.001 ^b, c^
EPDS at birth	3.48 (2.34)	9.70 (4.39)	8.36 (4.16)	Welch (2, 70.16) = 75.52, *p* < 0.001 ^a, b^
EPDS 3 weeks	4.48 (2.79)	10.71 (3.59)	11.97 (5.74)	Welch (2, 72.61) = 94.04, *p* < 0.001 ^a, b^
EPDS 6 weeks	3.33 (2.53)	8.60 (3.94)	11.72 (4.78)	Welch (2, 71.22) = 89.29, *p* < 0.001 ^a, b, c^
EPDS 9 weeks	2.71 (2.44)	6.56 (3.67)	11.56 (5.53)	Welch (2, 70.11) = 66.39, *p* < 0.001 ^a, b, c^
EPDS 12 weeks	2.30 (2.08)	6.11 (2.94)	12.61 (3.99)	Welch (2, 72.09) = 140.54, *p* < 0.001 ^a, b, c^
HCC T0 (pg/mg)	10.59 (15.20)	8.63 (8.46)	7.47 (5.21)	* F (2, 239) = 0.58, *p* = 0.943
HCC T1 (pg/mg)	5.54 (4.61)	4.99 (3.57)	6.21(5.32)	* Welch (2, 93.07) = 0.82, *p* = 0.444
HCNC T0 (pg/mg)	30.67 (30.07)	37.47 (33.99)	32.21 (33.54)	* F (2, 239) = 1.09, *p* = 0.337
HCNC T1 (pg/mg)	19.07 (13.24)	23.202 (15.13)	22.72 (16.79)	* F (2, 239) = 3.12, *p* = 0.046
HCC/HCNC ratio T0 (pg/mg)	0.60 (2.95)	0.40 (1.12)	1.30 (6.36)	* F (2, 239) = 2.04, *p* = 0.132
HCC/HCNC ratio T1 (pg/mg)	0.32 (0.19)	0.26 (0.19)	0.29 (0.13)	* F (2, 239) = 4.58, *p* = 0.011 ^a^

Note. EPDS: Edinburgh Postnatal Depression Scale; HCC: hair cortisol concentration; HCNC: hair cortisone concentration; T0: 1–6 days after birth; T1: 12 weeks postpartum. ^a,b,c^ Games–Howell-corrected significant difference (*p* < 0.05) between ^a^ ND and AD, ^b^ between ND and PPD and/or ^c^ between AD and PPD. * Tests were performed with log(2)-transformed glucocorticoid values.

**Table 2:** Spearman correlation of perceived stress and hair glucocorticoid concentrations.

	Sum of Perceived Stress		
	ND *n* = 141	AD *n* = 63	PPD *n* = 36
HCC T1	0.018 (0.417)	0.135 (0.291)	–0.139 (0.419)
HCNC T1	–0.153 (0.035)	0.318 (0.011)	–0.208 (0.225)
HCC/HCNC ratio T1	0.304 (0.000)	–0.126 (0.324)	0.104 (0.548)

Note. HCC = hair cortisol concentration; HCNC = hair cortisone concentration; ND = non-depressed; AD = adjustment disorder; PPD = postpartum depression; T1 = measurement occasion three months after delivery.

## Data Availability

The data presented in this study are available on request. They are not publicly available for the protection of the research participants’ privacy.

## Supplementary Materials

The following are available online at https://www.mdpi.com/article/10.3390/metabo11120815/s1, Table S1: Demographic information (means (M), standard deviations (SD) and frequencies (%)) of the study population, Figure S1: Log2-transformed mean concentration values (pg/mg) and standard errors (bars) of hair cortisol (HCC) and hair cortisone (HCNC) at both measurement time points (T0: third trimester of pregnancy and T1: 3 months postpartum) in all three groups.
