# Supplementary material for: Predicting Hair Cortisol and Cortisone Concentration in Postpartum Women through Repeated Measurements of Perceived Stress
Source: Metabolites. 2021 Nov 29;11(12):815. doi: 10.3390/metabo11120815 (PMC8707977; doi:10.3390/metabo11120815)
Supplement: Supplementary file 1 [file metabolites-11-00815-s001.zip › metabolites-1461438-supplementary.pdf]

Supplementary Information for

Predicting hair cortisol and cortisone concentration in postpartum women through repeated measurements of perceived stress

Lang J*, Stickel S*, Gaum PM, Habel U, Bertram J, Eickhoff SB, Chechko N

**Shared first authorship*

Results

Table S1. Demographic information (means [M], standard deviations [SD] and frequencies [%]) of the study population

	Non-depressed		Adjustment disorder		Postpartum depression		
	Mean (SD)	%	Mean (SD)	%	Mean(SD)	%	p*
**Annual income (Euro)**							
**< 20.000**		11.9		3.3		25.0	p = .005[c]
**20.000 – 50.000**		34.8		45.9		33.3	
**> 50.000**		53.3		50.8		41.7	
**Secondary education**							
**Lowest (<9 years)**		2.2		6.6		11.1	p = .039[b]
**Middle (~10 years)**		11.8		11.5		8.3	
**Highest (> 13 years)**		86.0		82.0		80.6	
**Married**		82.9		87.1		63.9	p = .013[c]
**Total number of children**	1.72 (0.85)		1.67 (0.88)		1.58 (0.73)		
**Week of gestation**	273.73 (11.22)		269.41 (18.52)		270.31 (17.93)		
**Birth mode**							
**Spontaneous**		54.6		39.7		58.3	
**Ventouse**		6.4		5.3		13.9	
**Elected C-section**		27.0		34.9		19.4	
**Emergency C-section**		12.1		15.9		8.3	
**Complication during pregnancy**		42.4		51.7		45.7	
**Complication during childbirth**		31.4		40.0		34.3	
**Child's birth weight**	3468.98 (615.95)		3418.17 (923.63)		3370.97 (815.22)		
**Child-related relocation to neonatal wards**		29.8		38.1		30.6	
**Prior depression (before pregnancy excl. PPD)**		10.3		31.7		47.2	p < .001[a,b]
**Prior PPD**		3.5		7.9		11.4	
**Family history of affective disorders**		19.9		47.6		36.1	p < .001[a]

Note. PPD = Postpartum depression

* only significant statistics are presented

[a,b,c] Post-hoc significant difference between [a] Non Depressed and Adjustment Disorder, [b] between Non Depressed and PPD and/or [c] between Adjustment Disorder and PPD.

Glucocorticoid concentration over the course of the third trimester and 12 weeks postpartum

The three groups were compared with regard to differences in the glucocorticoid concentration at T0 and T1 (see Suppl. Figure 1). For HCC and HCNC, no significant group differences were detected at either time point. However, in terms of the HCC-HCNC ration, the groups differed significantly at T1, with ND having a higher ratio than AD ($p = .014$, $d = 0.19$).

Within both groups, HCC and HCNC at T0 were compared to their respective values at T1. The dependent t-tests showed a significant mean difference between the HCC values at T0 and T1 for ND ($t(140) = 5.08$; $p < .001$, $d = .428$) and AD ($t(62) = 4.14$, $p = < .001$, $d = .522$), with the mean HCC values being lower three months after childbirth than in the third trimester of pregnancy in both groups. Furthermore, the HCNC value at T0 was significantly higher than at T1 both in ND ($t(140) = 4.63$, $p < .001$, $d = .390$) and AD ($t(62) = 3.11$; $p = .003$, $d = .392$). In the PPD group, no significant differences were found in HCC and HCNC levels between T0 and T1.

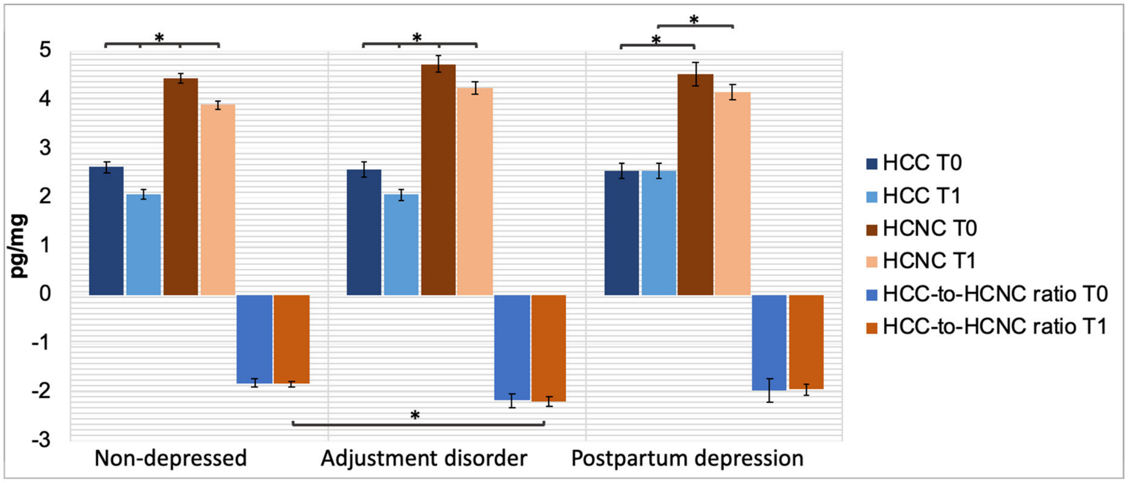

Figure S1. Log2-transformed mean concentration values (pg/mg) and standard errors (bars) of hair cortisol (HCC) and hair cortisone (HCNC) at both measurement time points (T0: third trimester of pregnancy, and T1: 3 months postpartum) in all three groups.
